# Aluminosilicate Zeolite EMM-28 Containing Supercavities Determined by Continuous Rotation Electron Diffraction

**DOI:** 10.1021/acs.inorgchem.2c00856

**Published:** 2022-07-11

**Authors:** Magdalena O. Cichocka, Allen W. Burton, Mobae Afeworki, Ross Mabon, Kirk D. Schmitt, Karl G. Strohmaier, Hilda B. Vroman, Michael A. Marella, Simon C. Weston, Xiaodong Zou, Tom Willhammar

**Affiliations:** † Department of Materials and Environmental Chemistry, Stockholm University, SE-106 91 Stockholm, Sweden; ‡ Corporate Strategic Research, ExxonMobil Research & Engineering Co., 1545 Route 22 East, Annandale, New Jersey 08801, United States

## Abstract

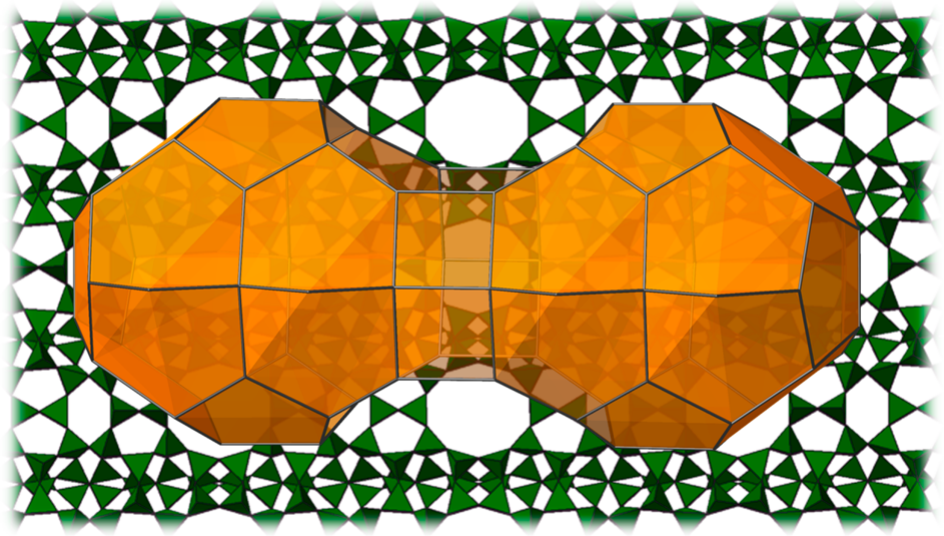

A new aluminosilicate zeolite, denoted EMM-28, has been successfully synthesized on a large scale using 1,1-(3,3-(1,3-phenylene)bis(propane-3,1-diyl))bis(1-methylpyrrolidinium) hydroxide as an organic structure directing agent (OSDA), which was scaled up to an ∼20 g scale with a yield of 77%. It crystallizes as thin plates (40–100 nm in thickness), and the corresponding powder X-ray diffraction (PXRD) pattern shows significant peak broadening which makes it insufficient for structure determination. Continuous rotation electron diffraction (cRED) data collected from 13 crystals were successfully used to solve and refine the structure of EMM-28. This illustrates that cRED data are capable of performing structure determination despite limited PXRD data quality. EMM-28 has a unique framework structure containing supercavities, >21 Å in size, connected by one-dimensional 10-ring channels. High-resolution transmission electron microscopy (HRTEM) confirmed the structure model. The structure of EMM-28 is related to several known zeolite structures with large cavities.

## Abstract 2

The properties of zeolites are largely determined by their atomically well-defined microporous structures of molecular dimensions. The new aluminosilicate zeolite, EMM-28, has a unique framework structure containing large supercavities connected by one-dimensional channels limited by 10 SiO_4_/AlO_4_ tetrahedra. The atomic structure of EMM-28 was determined using continuous rotation electron diffraction (cRED) data collected from 13 crystals. Despite limited powder X-ray diffraction data quality, the structure could be successfully refined using cRED data.

## Introduction

Zeolites are microporous crystalline materials with pores of molecular dimensions, which have found wide applications in catalysis, gas separation, and ion-exchange.^1^ The unique properties of these materials, e.g., shape selectivity and sorption capacity, are to a large extent determined by their structures. Zeolites have a large structural diversity, with pores denoted either by cavities with windows or channels that extend in one-, two-, or three dimensions. In order to understand the properties, predict possible applications, and design new synthesis routes for zeolites, it is of great importance to know their structures.

Three-dimensional electron diffraction (3D ED) has gained increasing attention in recent years for structure determination from submicrometer sized crystals. The recent developments of 3D ED techniques have been shown to be very powerful; almost complete 3D electron diffraction data can be obtained from an arbitrarily oriented crystal within a matter of minutes or less.^2−6^ A rapidly growing number of zeolite structures have been solved using the techniques,^7−11^ and the refinements have been shown to provide accurately refined structures.^12,13^

Many zeolites contain large cavities that are connected by smaller windows. These large internal cavities may act as chambers for bulky reaction intermediates. One example is faujasite with its large supercavities connected by 12-ring windows. Zeolite Y with the framework topology of faujasite (three letter code **FAU**) has been widely used as a catalyst for fluid catalytic cracking. Zeolites with the **MWW** topology contain large cavities interconnected by 10-ring windows.^14^ Other examples of zeolite structures containing cage structures are MCM-68^15^ (**MSE**), EU-1^16^ (**EUO**), SSZ-52^17^ (**SFE**), and ZEO-1.^18^

In order to explore zeolites with cavity-type structures, a family of diquaternary ammonium molecules was investigated as organic structure directing agents (OSDAs). Using the meta isomer, a novel crystalline aluminosilicate zeolite was discovered, denoted EMM-28. EMM-28 crystallizes as thin nanoplates of 40–100 nm in thickness. The powder X-ray diffraction (PXRD) pattern of EMM-28 was characteristic, but the peaks were not as sharp as one would expect from a highly crystalline material, potentially due to disorder or internal strain in the crystals. The lack of sharp reflections in the PXRD pattern prevented a successful structure determination from the PXRD data. In this study, we show that 3D electron diffraction methods can be used to successfully determine the structure of this novel zeolite material even though the material possesses a less than ideal PXRD pattern. The structure of EMM-28 shows an interesting pore structure with extra-large cavities accessible via 10-ring windows.

## Results and Discussion

**Figure 1:**
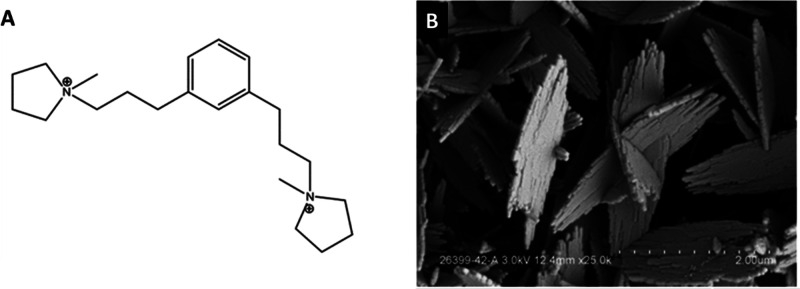
(A) 1,1-(3,3-(1,3-Phenylene)bis(propane-3,1-diyl))bis(1-methylpyrrolidinium) cation was used as the organic structure directing agent (OSDA) in the synthesis of EMM-28. (B) SEM micrograph of the EMM-28 crystal with a thickness of about 40–100 nm.

In the current study, we examined the structure directing effects of the meta analogue of an organic structure directing agent (OSDA) molecule that possess *N*-methylpyrrolidinium end groups connected to a phenyl core by a chain of 3 methylene groups. Using the 1,1-(3,3-(1,3-phenylene)bis(propane-3,1-diyl))bis(1-methylpyrrolidinium) hydroxide (see Figure 1A) as the OSDA, the new phase EMM-28 was successfully synthesized under conditions of high Si/Al ratios (>100).^19^ The representative large scale syntheses are reported in Table S1, where 72.7 g of colloidal silica (30 wt % silica) resulted in 16.9 g of calcined EMM-28, a yield of 77% based on silica. EMM-28 is obtained as thin plate-like crystals of 40–100 nm thickness by hydrothermal synthesis at 160 °C (Figure 1B). The total BET surface area and micropore volume of the EMM-28 product are 506 m^2^/g and 0.176 cm^3^/g, respectively. The uptakes of *n*-hexane (0.089 g/cm^3^) and 2,3-dimethylbutane (0.077 g/cm^3^) are both very fast, while that for 2,2-dimethylbutane (0.044 g/cm^3^) is slow (see isotherm in Figure S1). This adsorption behavior is indicative of a zeolite with medium pores. The magnitude of the capacity indicates either that the zeolite is multidimensional or that it is a one-dimensional pore zeolite with large side pockets–like the **EUO**-type zeolites (ZSM-50 or EU-1).

**Figure 2:**
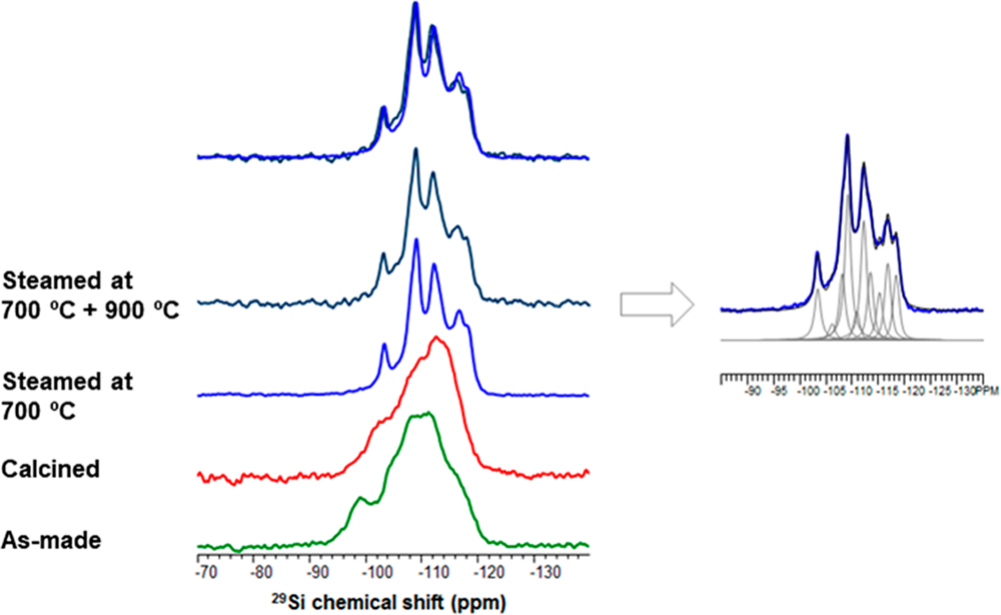
^29^Si MAS NMR spectra of EMM-28. As-made material is shown in green as well as after different stages of treatment: calcined (orange), steamed at 700 °C (blue), and further steamed at 900 °C (dark blue). The sample survived the steaming at 900 °C (superimposed spectra at top). Deconvolution of the data from the 700 °C steamed sample shows at least ten unique T-sites (right).

Solid state ^29^Si NMR of EMM-28 was performed on samples after different stages of treatment. As expected for an all-silica material prepared from a hydroxide medium, the spectrum of as-made EMM-28 shows broad peaks containing both Q3 and Q4 species. The Q3 peaks are reduced after calcination, but the spectrum remains broad. After steaming the sample to 700 and 900 °C, there is a sharpening of the peaks as the remaining internal silanols anneal, and the environment of the individual T sites becomes more homogeneous. Deconvolution of the spectrum indicates that there are at least 10 symmetry-independent T sites in the structure (Figure 2).

The PXRD pattern shows significant peak broadening, which might be due to disorder or the very thin plate-like morphology of the crystals (Figures 1B, 1A and Figure S2). The peak broadening in combination with the large unit cell parameters of the material (14–42 Å) gives rise to significant peak overlap, which hampers an accurate intensity integration, see Figure S3 for a comparison between the experimental PXRD pattern of EMM-28 and simulated pattern from the final structure. Accurately measured intensities are essential in order to use the PXRD pattern for *ab initio* structure determination. Hence, we were directed toward other methods to determine the structure. Similarities in the PXRD pattern of EMM-28 with that of **EUO** were observed (Figure S4). The PXRD pattern of EMM-28 could be indexed with a unit cell where the *a* and *b* parameters are similar to those of **EUO** but the *c* parameter is approximately doubled. This suggests that the structural model for EMM-28 may be derived from the layers present in the **EUO** structure.

In order to solve the structure of EMM-28, continuous rotation electron diffraction (cRED) data were collected from a calcined sample (Figure S5, Table S2). The crystal tilting range was 52.4°, and the data acquisition time was only 1 min. The cRED data could be indexed using a face-centered unit cell, with the lattice parameters *a* = 14.11 Å, *b* = 22.59 Å, *c* = 41.72 Å, α = 89.07°, β = 89.99°, and γ = 88.85°, indicating that the crystal might be orthorhombic. From the reconstructed 3D reciprocal lattice, the following reflection conditions were deduced: *hkl*: *h* + *k* = 2*n*, *h* + *l* = 2*n*, *k* + l = 2*n*; 0*kl*: *k*,*l* = 2*n*; *h*0*l*: *h, l* = 2*n*; *hk*0: *h*,*k* = 2*n*. These are consistent with space groups *F*222 (No. 22), *Fmm*2 (No. 42), and *Fmmm* (No. 69). Considering most of the zeolite frameworks in the IZA database^20^ are centrosymmetric, space group *Fmmm* was chosen for structure determination. Intensities of reflections were extracted using the software XDS^21^ (Table S2). Although the data resolution was relatively low (1.3 Å) and data completeness was low (47.8%), the framework structure could be solved in a straightforward manner using the program *Focus*(22,23) (Table S2). All 10 T atoms (T = Si, Al) were found, and O atoms were added in the expected positions between the T atoms. The low resolution and low completeness of the cRED data however hampered a successful structure refinement at this point.

**Figure 3:**
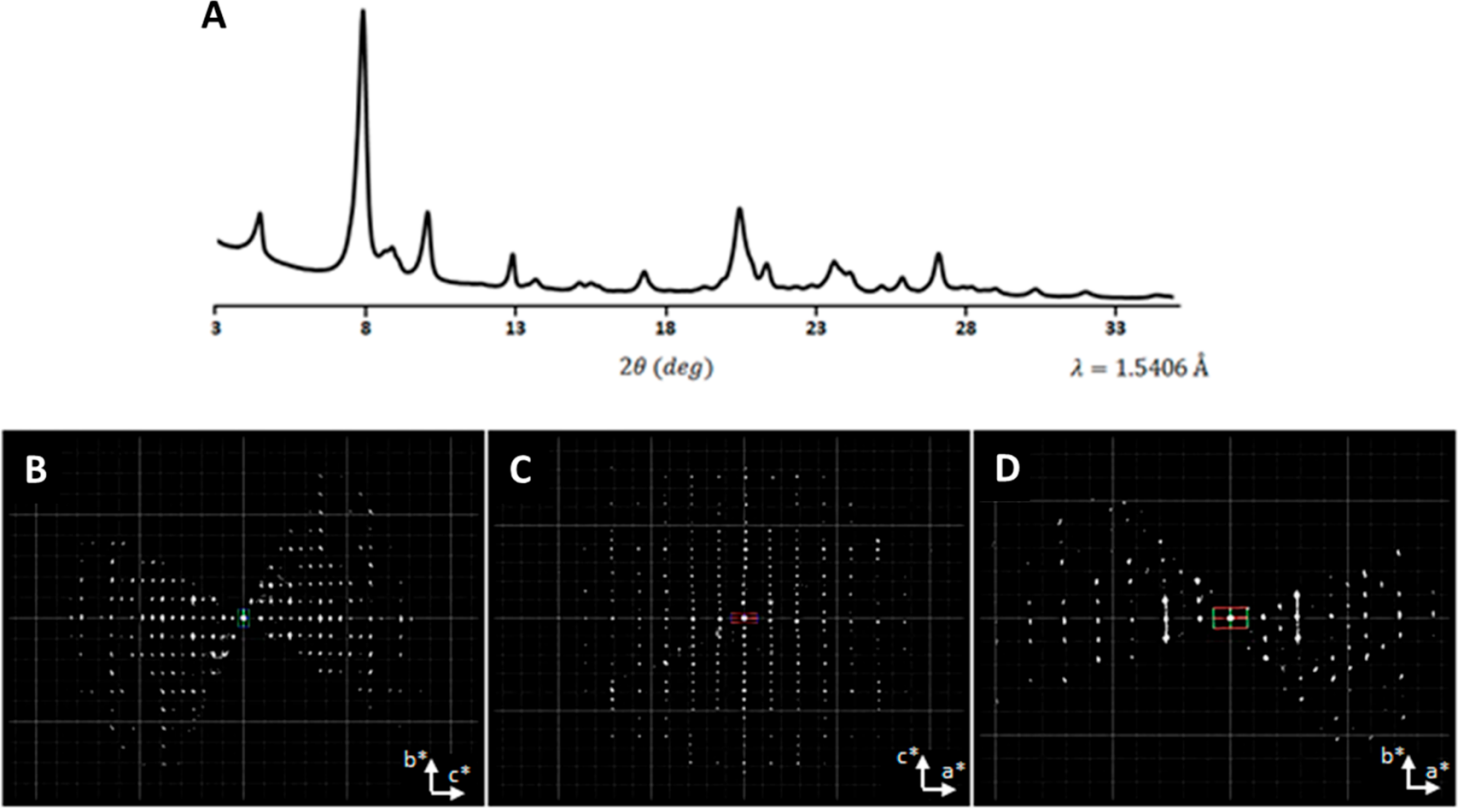
(A) Experimental PXRD data of calcined EMM-28. (B–D) Two-dimensional slices cut from the reconstructed three-dimensional lattice showing the (B) 0*kl*, (C) *h*0*l*, and (D) *hk*0 planes. Diffuse scattering is observed, shown as streaks along the b*-axis in (D).

In order to achieve higher quality data sets suitable for structure refinement and evaluate the occurrences of the streaks observed in the cRED data,^10^ we collected more cRED data on 13 different crystals using the software *Instamatic* which allows crystal tracking during data collection so that a larger rotation range can be achieved.^24^ The crystal tilting ranges were between 64.56° and 126.4°, and the data acquisition time for each crystal was 2.3–4.6 min. All the data sets could be indexed with the same face-centered orthorhombic unit cell as above, and some diffusely scattered streaks could be observed along the b*-direction, see Figure 3B–D. The intensities were extracted in space group *Fmmm* using *XDS*. A summary of data collection and data reduction statistics of all 13 data sets is given in Table S3. The structure of EMM-28 was solved with all of the following programs: *SHELXT*,^25^*SIR2014*,^26^ and *Focus*.^22,23^ The framework structure determined was identical to the one found initially using *Focus*.

**Figure 4:**
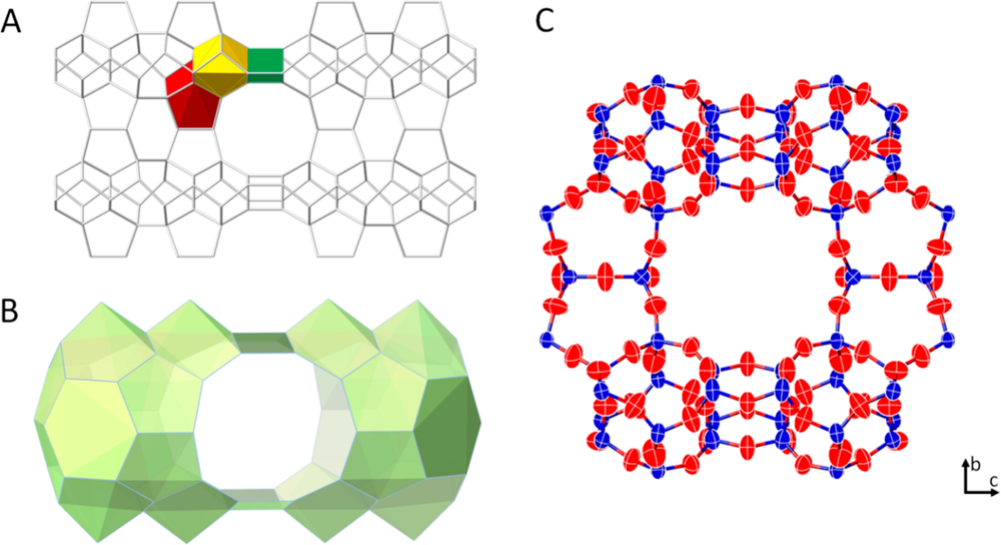
(A) EMM-28 with its building units highlighted: *non* (red) and *cas* (yellow) and a double layer constituted by chains of TO_4_ tetrahedra running along the *a*-axis (green). (B) A supercavity [4^4^5^12^6^20^10^2^] with two side pockets connected to a 10-ring channel. (C) A fragment of the refined structure of EMM-28 viewed along the *a*-axis showing anisotropic atomic displacement parameters for T-atoms (*T* = Si/Al) and O-atoms.

In order to obtain the best possible combination of data sets, an automated script was created to evaluate the impact of merging all possible combinations of cRED data sets for the final structure refinement. All possibilities were evaluated in an automated procedure. The best result was found to be a sequence of three data sets (3, 9, and 13: see Table S3 and Table 1) that were merged and scaled using the program *XSCALE* to obtain a higher completeness.^27^ The merged data set was used for structure refinement using *SHELXL* (Table 1).^28,29^ Similarity restraints were applied to all T–O bonds and O–O distances (and correspondingly the O–T–O angles) to keep the geometry reasonable. They were refined to 1.594(17) Å for the T–O bond distance and 109.5(1.7)° for the O–T–O angle (Table 2) using the lattice parameters determined from PXRD data (*a* = 13.946(3) Å, *b* = 22.580(5) Å, *c* = 40.402(8) Å). One of the oxygen atoms is positioned in a special position at an inversion center, which results in a T–O–T angle of 180°. In the absence of such restraints, the O–T–O angles would refine to values that were too low. All T and O atoms were refined anisotropically. Rigid-bond restraints were introduced to all framework atoms to keep the anisotropic displacement parameters (ADPs) reasonable (Figure 4C). In a recent publication, it has been shown that physically meaningful anisotropic ADPs that indicate the quality of the data can be obtained from cRED data.^10^ In the final stage of the refinement, an extinction coefficient (EXTI) was introduced. This reduced the *R*_1_ value. The refinement converged with an *R*_1_ value of 16.74% (Table 1).

**Table 1:** Crystallographic Details for the Refinement of EMM-28 Using the Merged Data Set

	merged data set
chemical formula (refined)	Si_224_O_448_
space group	*Fmmm* (69)
*a* (Å)	13.946(3)
*b* (Å)	22.580(5)
*c* (Å)	40.402(8)
volume (Å^3^)	12723(5)
resolution (Å)	1.03
total no. reflections	13664
no. unique reflections (all)	1697
no. unique reflections (*F*_o_ > 4σ(*F*_o_)	1055
*R*_*int*_	0.2275
data redundancy	8.05
completeness (%)	100
parameters	218
no. of restraints	340
*R*_1_ for all reflections	0.1945
*R*_1_ for *F*_o_ > 4σ(*F*_o_)	0.1674
GOOF	1.233

**Table 2:** Framework Bond Angles and Distances of the Refined EMM-28 Model Using cRED Data

	nominal value	min	max	average
T–O (Å)	1.61	1.565	1.636	1.594 (17)
O–T–O (deg)	109.5	105.1	112.1	109.5 (1.5)
T–O–T (deg)	145.0	140.9	180.0	157(12)

The framework structure of EMM-28 can be described as a set of interconnected *non* and *cas* units connected with a double layer constituted by chains of TO_4_ tetrahedra running along the *a*-axis (Figure 4A). It contains a one-dimensional straight 10-ring channel system along the *a*-axis. In addition, the structure contains supercavities ([4^4^5^12^6^20^10^2^]) with two side pockets which are connected to the 10-ring channels. The 10-ring channels have an effective size of 4.87 × 5.74 Å (assuming an oxygen van der Waals radius of 1.35 Å) (Figure S6).

**Figure 5:**
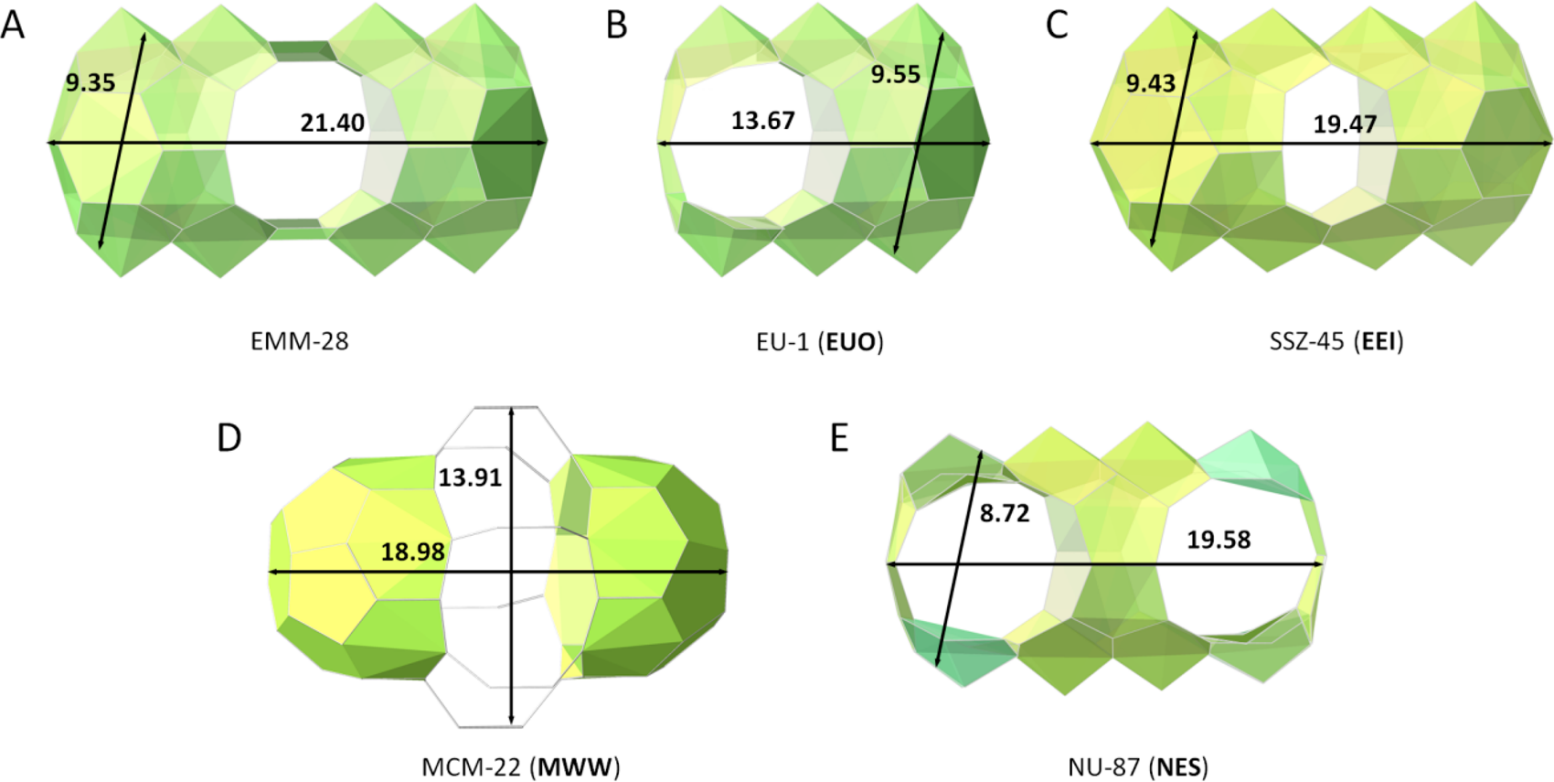
Supercavity connected by one 10-ring channel in (A) EMM-28, (B) EU-1, and (D) MCM-22, two 10-ring channels in (E) NU-87, and 8-ring channels in (C) SSZ-45. All van der Waals oxygen atom diameters of 2.7 Å have been subtracted.

The framework structure of EMM-28 is closely related to both EU-1^30^ (**EUO**) as well as NU-87^31^ (**NES**). The structure of EMM-28 is built from the same layer as in the **EUO** framework. By translating every second layer in the *ab*-plane of the **EUO** framework by 1/2*a*, the structure of EMM-28 is formed. This operation introduces a doubling of the unit cell along the *c*-axis. The relationship between **EUO**, **NES**, and **NON** zeolites has previously been described beautifully by Zanardi et al.^32^ All the above-mentioned materials contain internal cavities in their structures, as depicted in Figure 5. The dimensions of these cavities are closely related. The supercavity of EMM-28 is accessible via 10-ring channels running along [100], with free dimensions of 4.87 × 5.74 Å. The supercavity has two side pockets (each with a width of 9.35 Å), which are located on each side of the 10-ring channel and are connected by the channel. A similar situation has been found in other zeolites such as the structurally related NU-87 (**NES**) with double cavities defined by 10-ring channels (4.98 × 6.49 Å) and its length of 19.58 Å and MCM-22^14^ (**MWW**) with an inner supercavity of 8.44 Å in diameter and a length of 18.98 Å. The supercavity in SSZ-45 (**EEI**) is also similar to that of EMM-28, with two similar side pockets connected by a smaller channel (8-ring, 4.50 × 3.05 Å). Unlike the above examples, EU-1 (**EUO**) has only one side pocket connected to a 10-ring channel (4.84 × 6.12 Å), which results in a supercavity with a shorter length (13.67 Å) compared to that in EMM-28. It should be mentioned that these different zeolite structures were synthesized by using different OSDAs, which may play important structure directing roles in the formation of different cavities, see Table S4.

**Figure 6:**
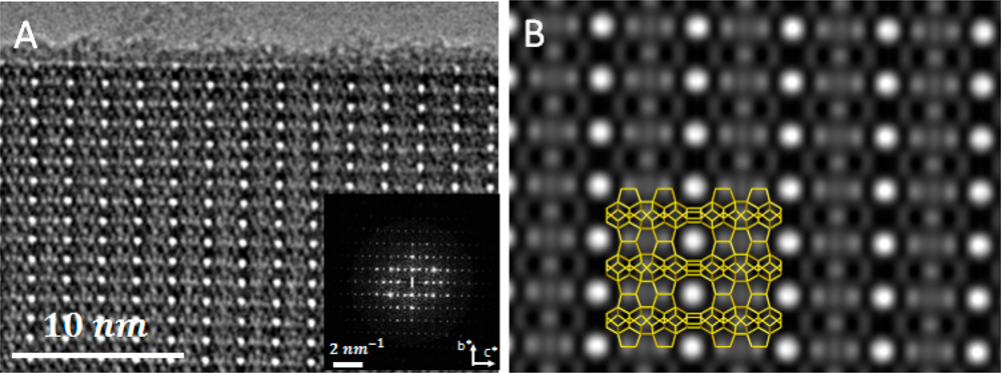
(A) Structure projection image along the *a*-axis, reconstructed using *QFocus* software from a through-focus series of 20 HRTEM images, showing the 10-ring channels in the structure of EMM-28. An inset shows Fourier transform (FT) from the image in (A). (B) Lattice averaged and symmetry-imposed projected potential map obtained by crystallographic image processing of the image in (A). The plane group symmetry *pmm* has been imposed.

High-resolution transmission electron microscopy (HRTEM) was performed to reveal more details about the pore structure in EMM-28 crystals. In order to observe the 10-ring channels which are parallel to the thin crystal plates (along the *a*-axis), ultramicrotomy was applied to prepare cross sections of the plate-like crystals. A through-focus series of HRTEM images with a defocus step of 53.3 Å were acquired to minimize the focusing and acquisition time. The structure projection image (Figure 6) was reconstructed using the program *QFocus*.^33^ The 10-ring channels along the *a*-axis are clearly observed in the image. The *b*-axis is perpendicular to the plate-like crystals and the 10-ring channels.

## Conclusions

A new aluminosilicate zeolite EMM-28 has been successfully synthesized at a large scale using 1,1-(3,3-(1,3-phenylene)bis(propane-3,1-diyl))bis(1-methylpyrrolidinium) hydroxide as the organic structure directing agent (OSDA). The structure was determined *ab initio* based on continuous rotation electron diffraction data. Despite the low quality of the PXRD pattern, the quality of the continuous rotation electron diffraction data is high enough to solve and refine the structure, which resulted in a chemically reasonable bond geometry and atomic displacement parameters. Local information was studied from reconstructed structure projection images based on through-focus series of HRTEM images. These further confirmed the refined structural model. This structure determination of EMM-28 shows the feasibility of 3D ED as a tool to study materials with PXRD patterns with significant peak overlap, which extends beyond zeolites into MOFs as well as other inorganic materials. EMM-28 has a novel zeolite framework with a one-dimensional 10-ring channel system with mesoporous cavities directed by the OSDA. The structure of EMM-28 is related to several known zeolite structures with large cavities and has one of the largest supercavities observed so far in zeolites.

## Experimental Section

### Synthesis

Hydrothermal syntheses were performed at 160 °C for 28 days in sealed Parr reactors with volumes of 23 and 60 mL and in a 300 mL overhead stirred autoclave. 1,1-(3,3-(1,3-Phenylene)bis(propane-3,1-diyl))bis(1-methylpyrrolidinium) hydroxide was used as the organic structure directing agent (OSDA). The representative large scale syntheses are reported in Table S1. The OSDA produces EMM-28 when the synthesis is performed with a high Si/Al ratio (>100).

### ^29^Si Solid State NMR

Solid state ^29^Si NMR spectra of EMM-28 were recorded on a Varian InfinityPlus 500 spectrometer. It shows the content of Si species of different coordinations. As expected for the spectrum of an all-silica material prepared from a hydroxide medium, the as-made spectrum contains a large fraction of Q3 species. After calcination, there is a reduction in the density of Q3 sites, but the spectrum remains broad. After steaming the sample to 700 and 900 °C, there is a sharpening of the peaks, as the remaining internal silanols anneal and the environment of the individual T sites becomes more homogeneous, see Figure 2 for the ^29^Si NMR spectrum of EMM-28 after different treatments.

### Calcination

The as-made EMM-28 was heated inside a muffle furnace from ambient temperature to ca. 400 °C at a heating rate of ca. 4 °C/min under a nitrogen atmosphere, then heated to ca. 600 °C at ca. 4 °C/min in air, and maintained at ca. 600 °C in air for about 2 h. The calcined product was then measured with nitrogen physisorption, and the data were analyzed by the t-plot method, according to the method of Lippens et al.^34^

### Adsorption

A sample of calcined EMM-28 was tested for nitrogen sorption. The material was thermally treated at about 500 °C for a time sufficient to substantially dehydrate the materials and/or to remove any adsorbed species prior to doing the sorption test.

### X-ray Powder Diffraction

The X-ray powder diffraction data reported herein were collected on a PANalytical X-Pert Pro diffraction system, equipped with an XCelerator detector, using copper K_α1_ radiation and a fixed 0.25 degrees divergence slit. The diffraction data were recorded by step-scanning at 0.017 degrees of two-theta and a counting time of about 2 s for each step.

### Continuous Rotation Electron Diffraction (cRED)

A small amount of EMM-28 was crushed in the mortar and then dispersed in ethanol in an ultrasonic bath for 1–2 min. A droplet of the suspension was transferred onto a carbon-coated copper grid.

The data set of EMM-28 used for the initial structure solution was collected by using continuous Rotation Electron Diffraction (cRED)^8,35−37^ on a JEOL JEM2100 transmission electron microscope (TEM) at room temperature and 200 kV. A single-tilt tomography sample holder was used for the data collection, which can tilt from −70° to +70°. Electron diffraction frames were recorded on a Timepix camera in selected area electron diffraction (SAED) mode with the spot size 3 and the camera length of 50 cm. The cRED data sets used for the structure refinement were collected using *Instamatic*,^10,24^ which simplifies the data collection procedure by including semiautomated crystal tracking and results in more reliable, complete, and reproducible data collections.

Data reduction for all crystals was performed using the *XDS* software.^21^

### Through-Focus Series of Images

A sample for HRTEM imaging was prepared by ultramicrotomy. This is advantageous since it is desired to study the cross section of the plate-like crystals. The through-focus series of HRTEM images with a defocus step of 53.3 Å were acquired on a JEOL JEM2100F TEM at 200 kV. The structure projection images were reconstructed by program *QFocus*(33) using a contrast transfer function compensation algorithm.

## Supporting Information Available

Summary of synthesis, gas sorption isotherm, powder X-ray diffraction data, electron diffraction details, refinement statistics, and structural analysis (PDF)

## Accession Codes

CCDC 2159049 contains the supplementary crystallographic data for this paper. These data can be obtained free of charge via www.ccdc.cam.ac.uk/data_request/cif, or by emailing data_request@ccdc.cam.ac.uk, or by contacting The Cambridge Crystallographic Data Centre, 12 Union Road, Cambridge CB2 1EZ, UK; fax: +44 1223 336033.
