# Supplementary material for: Aluminosilicate Zeolite EMM-28 Containing Supercavities Determined by Continuous Rotation Electron Diffraction
Source: Inorg Chem. 2022 Jul 11;61(29):11103–9. doi: 10.1021/acs.inorgchem.2c00856 (PMC9490810; doi:10.1021/acs.inorgchem.2c00856)
Supplement: Supplementary file 1 — ic2c00856_si_001.pdf [file ic2c00856_si_001.pdf]

Supporting information

An aluminosilicate zeolite EMM-28 containing super-cavities determined by continuous rotation electron diffraction

Magdalena O. Cichocka,[a] Allen W. Burton,[b,*] Mobae Afeworki,[b] Ross Mabon,[b] Kirk D. Schmitt,[b] Karl G. Strohmaier,[b] Hilda B. Vroman,[b] Michael A. Marella,[b] Simon C. Weston,[b] Xiaodong Zou,[a] Tom Willhammar,[a,*]

[a] Department of Materials and Environmental Chemistry, Stockholm University, SE-106 91 Stockholm, Sweden

[b] Corporate Strategic Research, ExxonMobil Research & Engineering Co., 1545 Route 22 East, Annandale, New Jersey 08801, USA

Corresponding Authors:
* tom.willhammar@mmk.su.se, allen.w.burton@exxonmobil.com

Table S1. *Larger-scale syntheses performed in autoclaves. All syntheses were carried out at 160 °C with the meta OSDA.*

**Scale**	**Si source**	**Al source**	**EMM-28 seeds (Y/N)**	**Si/Al**	**H_2O/Si**	**OH/SiO_2**	**Product**	**Days**
23 ml	Ludox LS-30	Fumed Al_2O_3	Y	500	30	0.30	EMM-28<br><br>(slight amorphous)	14
23 ml	Ludox LS-30	Fumed Al_2O_3	Y	1000	30	0.30	EMM-28	14
23 ml	Ultrasil	Fumed Al_2O_3	Y	100	30	0.30	EMM-28	14
23 ml	Ludox LS-30	Fumed Al_2O_3	Y	500	25	0.20	EMM-28	17
23 ml	Ludox SL-30	$Al(NO_3)$	Y	1000	30	0.30	EMM-28	15
23 ml	Ludox SL-30	$Al(NO_3)$	N	500	30	0.30	EMM-28	22
23 ml	Ludox SL-30	MS-25	Y	500	30	0.30	EMM-28 (small unknown impurity)	14
60 ml stirring autoclave	Ludox SL-30	MS-25	Y	500	30	0.30	EMM-28	14
1st synthesis post high throughput discovery (10 ml)	Ludox LS-30	MS-25	N	500	30	0.30	EMM-28	28
1nd synthesis post high throughput discovery (10 ml)	Ludox LS-30	MS-25	N	500	30	0.30	EMM-28	28
300 ml stirred autoclave	Ludox LS-30	$Al(NO_3)$	Y	500	30	0.30	EMM-28	14
300 ml stirred autoclave	Ludox LS-30	$Al(NO_3)$	Y	500	30	0.30	EMM-28	14

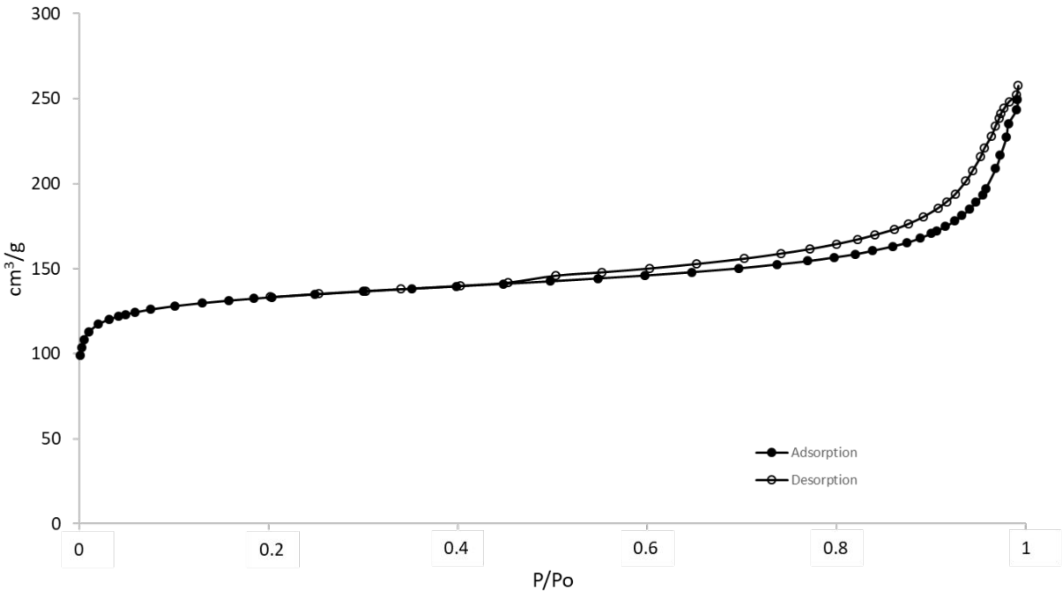

Figure S1. *Nitrogen sorption isotherm from EMM-28. The solid symbols are adsorption data points and the hollow symbols are desorption data points.*

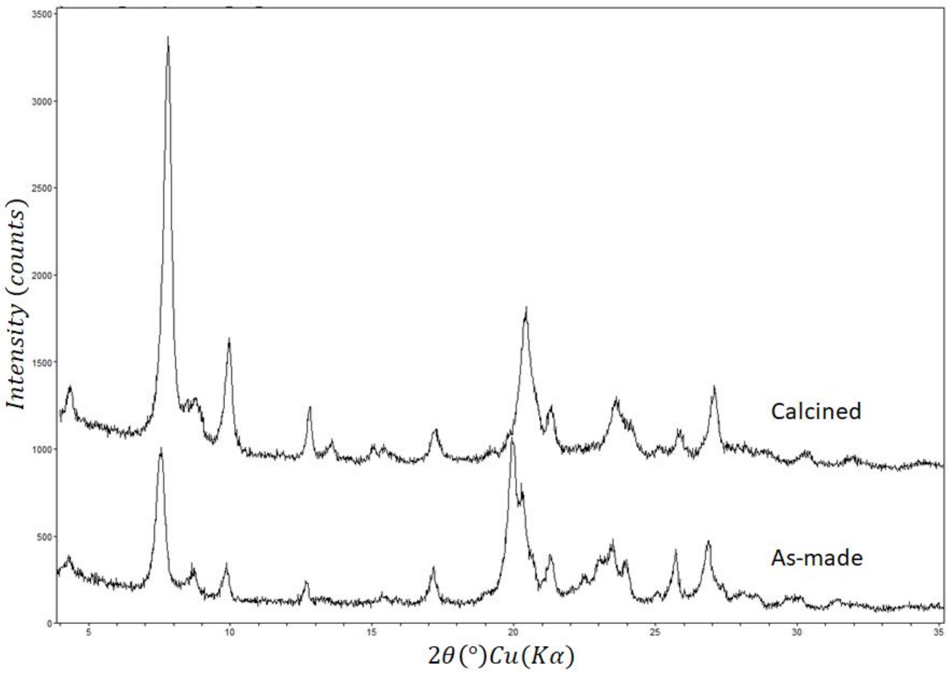

Figure S2. *PXRD pattern of EMM-28 before and after calcination (*$\lambda = 1.5406$ Å).

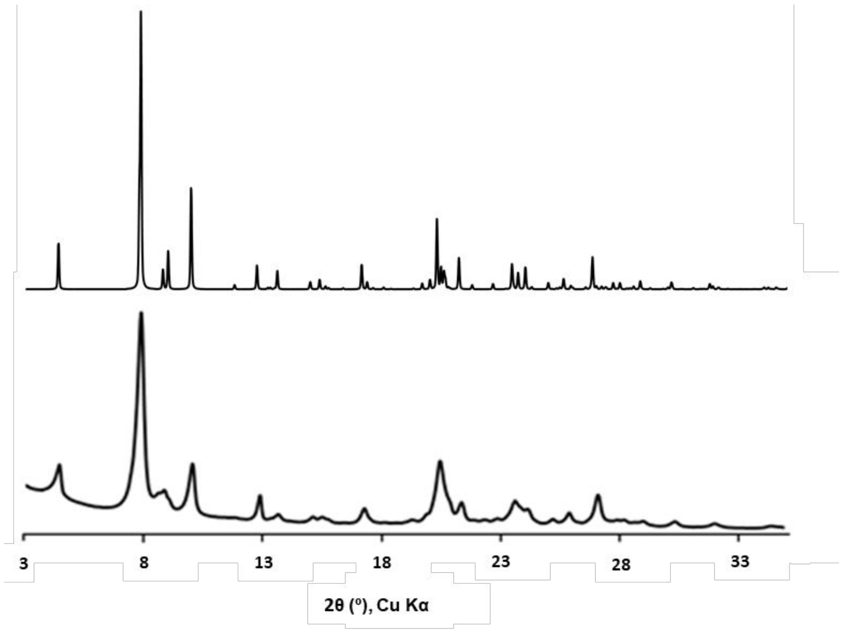

Figure S3. *Experimental PXRD pattern from EMM-28 (bottom) and simulated pattern from the final refined structure of EMM-28 shows the significant peak broadening of the experimental pattern.*

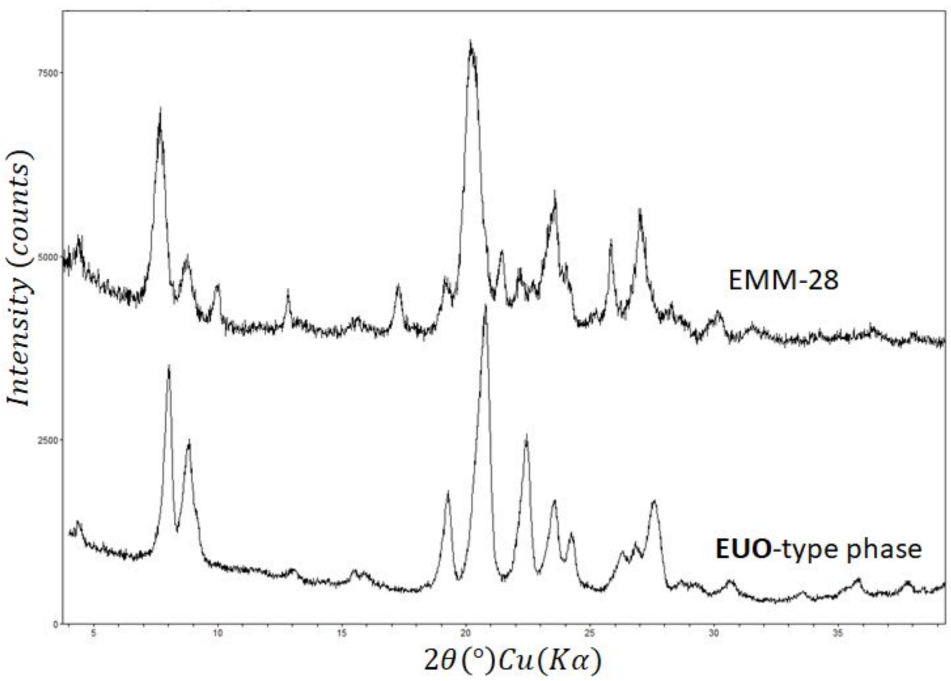

Figure S4. *Comparison of the PXRD patterns of as-made EMM-28 and an as-made* ***EUO****-type zeolite* $(\lambda = 1.5406\ \text{Å})$.

The initial structural model was obtained from the cRED data using the program Focus[1,2]. After 100 000 trials, the most frequent result was found 40% of the time, which is significant enough to indicate correspondence to the correct framework structure.

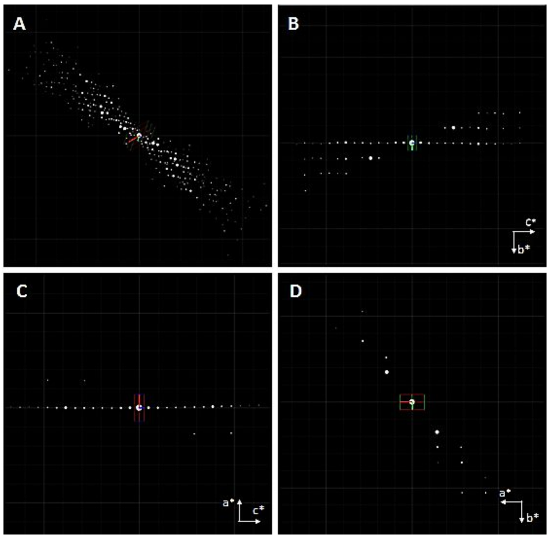

Figure S5. *(A) Reconstructed 3D reciprocal lattice of EMM-28 from the 3D RED data. (B - D) Two-dimensional slices cut from the reconstructed three – dimensional lattice showing the (B) (0kl), (C) (h0l) plane and (D) (hk0) plane.*

Table S2. *Experimental parameters for the cRED data collection of EMM-28 and data processing details using XDS.*

Parameters	Crystal
Tilt range (°)	52.4 (-37.1 to 15.3)
Goniometer tilt step (°/frame)	0.13067
Total data collection time (min)	1
No. of frames	376
Resolution (Å)	1.3
Completeness (%)	47.8
No of unique reflections	430
No. of observed reflections	1045
I/σ	8.12
R_{meas} (%)	8.4
R_{obs} (%)	6.4
R_{exp} (%)	6.5

Table S3. *Experimental details for the cRED data collection of 13 datasets of EMM-28 by using Instamatic software (λ = 0.02508 Å (200 keV), exposure time/frame =0.6 s, spot size=1, camera length = 250 mm) and data processing details using XDS for 13 datasets of EMM-28.*

	**Dataset 1**	**Dataset 2**	**Dataset 3***	**Dataset 4**	**Dataset 5**	**Dataset 6**	**Dataset 7**	**Dataset 8**	**Dataset 9***	**Dataset 10**	**Dataset 11**	**Dataset 12**	**Dataset 13***
Oscillation angle (°)	0.2851	0.2833	0.281	0.2834	0.2869	0.2834	0.286	0.2824	0.2826	0.2853	0.2846	0.2857	0.2843
Tilt range(°)	- 38.29 to 60.07 (98.36)	- 57.79 to 67.44 (125.23)	- 70.17 to 33.80 (103.97)	- 59.46 to 66.94 (126.4)	- 43.80 to 20.76 (64.56)	- 59.92 to 66.48 (126.4)	- 46.28 to 52.69 (98.97)	- 18.49 to 70.17 (88.66)	- 69.26 to 23.99 (93.25)	- 35.82 to 52.34 (88.16)	- 18.79 to 60.32 (79.11)	- 49.61 to 62.09 (111.7)	- 19.09 to 60.22 (79.31)
Frames used[1]	330	435	346	440	218	396	267	308	313	293	269	375	261
Total acquisition time (min)	3.6	4.5	3.8	4.6	2.3	4.6	3.5	3.2	3.4	3.2	2.8	4.0	2.9
Resolution (Å)	1.00	1.00	1.00	1.00	1.00	1.00	0.80	1.00	0.90	1.00	1.08	1.00	1.00
No. of total reflections	5668	7640	5770	7755	3634	6657	9075	5223	6365	4656	3418	6378	3545
No. of unique reflections	1607	1471	1415	1621	1270	1581	2310	1694	2015	1344	999	1806	1576
Completeness (%)	85.7	78.4	74.4	84.7	68.8	86.3	64.8	86.3	81.3	73.1	68.0	96.9	83.7
I/σ	0.92	2.75	4.05	2.42	3.14	4.22	1.58	3.32	3.37	1.15	0.88	1.5	1.76
Rmeas (%)	57.5	29.0	16.3	21.9	17.7	16.3	27.0	16.2	16.8	39.3	61.8	43.5	26.2
Robs(%)	49.4	25.4	14.0	19.0	14.2	14.1	23.4	13.2	14.2	33.2	51.0	37.0	20.8
Rexp (%)	48.1	25.5	14.6	21.0	15.2	15.2	24.1	13.7	14.9	40.3	48.2	34.9	21.6

[*]Datasets used for the final structure refinement

The distances and angles of the refined structure of EMM-28 were analyzed by PLATON software. The anisotropic displacement parameters (ADPs) were validated using the Hirshfeld Rigid-Bond test in PLATON software,[3] indicating that the quality of the examined datasets is good and the refined ADPs are sensible.

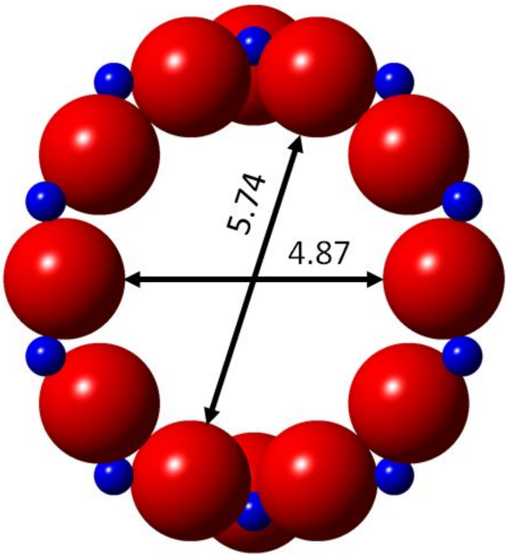

Figure S6. *Pore opening of the 10-ring channel along the a-axis. All O···O distances are in Ångstrom where an oxygen diameter of 2.70 Å has been subtracted.*

Table S4. *Comparison of EMM-28, EU-1, SSZ-45, MCM-22, and NU-87.*

	EMM-28	EU-1 (**EUO**)	SSZ-45 (**EEI**)	MCM-22 (**MWW**)	NU-87 (**NES**)
Space group, Unit cell parameters	*Fmmm*(69) *a*=13.946(3) Å *b*=22.580(5) Å *c*=40.402(8) Å	*Cmme*(67) *a*= 13.6950 Å *b*= 22.3260 Å *c*= 20.1780 Å	*Fmm2*(42) *a*= 13.7195 Å *b*= 35.2245 Å *c*= 22.1362 Å	*P*6/*mmm*(191) *a*= 14.2080 Å *b*= 14.2080 Å *c*= 24.9450 Å	$P12_1/c1$(14) *a*= 14.3240 Å *b*= 22.3760 Å *c*= 25.0920 Å
No. of unique T-atoms	10 (Si/Al)	10 (Si/Al)	10 (Si)	8 (Si)	7 (Si)
Chain	Double zigzag chain II *a*	Single zigzag chain II *c*	Single zigzag chain II *a*	not applicable	Single zigzag chain II *a*
Composite building units	*non, cas*	*non, cas*	*non, cas*	*d6r, mel*	*cas, non, ton*
Channel system	1D	1D	1D	2D	2D
Channel size	10-ring *II a*	10-ring *II a*	8-ring *II a*	10-ring *II b*	10-ring *II b*
OSDA	1,1-(3,3-(1,3-phenylene)bis(propane-3,1-diyl))bis(1-methylpyrrolidinium) hydroxide (meta isomer)	Hexamethonium (HM) Dibenzyldimethylammonium (DBDMA)	N-cyclopentyl-DABCO	Trimethyladamantammonium ($TMAda^+$)	Decamethonium

References

1 R. W. Grosse-Kunstleve, L. B. McCusker and C. Baerlocher Powder Diffraction Data and Crystal Chemical Information Combined in an Automated Structure Determination Procedure for Zeolites *J. Appl. Crystallogr.*, **1997**, 30, 985–995.

2 S. Smeets, L. B. McCusker, C. Baerlocher, E. Mugnaioli and U. Kolb Using FOCUS to solve zeolite structures from three-dimensional electron diffraction data *J. Appl. Crystallogr.*, **2013**, 46, 1017–1023.

3 A. L. Spek Structure validation in chemical crystallography *Acta Cryst D*, **2009**, 65, 148–155.
